# Quantifying the visual information sourced from melanopsin photoreceptors in mouse LGN field responses

**DOI:** 10.1186/1471-2202-12-S1-P226

**Published:** 2011-07-18

**Authors:** Sohail Siadatnejad, Timothy M Brown, John Gigg, Hugh D Piggins, Robert J Lucas, Marcelo A Montemurro

**Affiliations:** 1 Faculty of Life Sciences, University of Manchester, Manchester, M13 9PT, UK

## 

Melanopsin photoreceptors make a third type of photoreceptor along with rods and cones in human and mouse. Until recently melanopsin was thought to participate only in subconscious responses to light (such as pupillary reflexes and regulating the circadian rhythm) and not in image-forming visual responses. Recently, it has been shown that melanopsin derived signals are also widespread in image-forming visual pathways of mice [1]. The goal of this study is to quantify their contribution in the visual pathway. We used information theoretic measures [2,3] on field potentials [3] to quantify the amount of information that is originated in melanopsin photoreceptors. The continuous field potentials were recorded from lateral geniculate nucleus (LGN) of transgenic mice (Opn1mw^R^) [1] using multichannel electrodes.

The role of melanopsin was quantified by estimating the information [2] found in LGN responses, about the intensity of constant blue (460nm) and red (655nm) light (7 levels of irradiance were used). The intensities of these two lights were carefully matched to provide equal stimulation of the red-sensitive cones of Opn1mw^R^ animals and to control for the influence of rod photoreceptors. Since the long wavelength (red) light was essentially invisible to melanopsin, subtracting the amount of information in 655nm stimuli from the 460nm should reveal the visual information that is sourced from melanopsin photoreceptors in each recording channel. To investigate the local continuous response signals, the power and phase of recorded field signals were examined at various frequency bands and time points. For each spectrotemporal component of the responses, information about the intensity of light was estimated [1,3] separately for blue and red lights for each recording channel.

**Figure 1:**
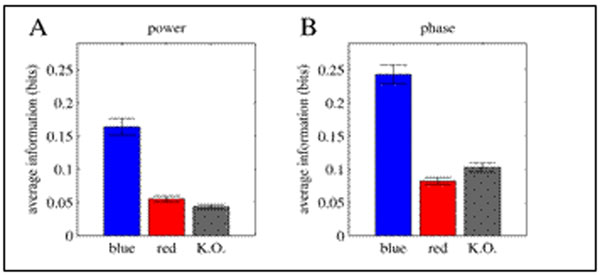
Quantification of melanopsin information. Information estimated for responses to blue and red are averaged and shown with SEM (averaged over total 372 channels that lay in LGN for 17 animals). Information in response to blue light is on average greater than the information estimated in responses of the same units to red light. Panel A shows this information comparison for power and B for phase of field potentials recorded in each channel. The power and phase response values were discretized into four levels for information estimations. **A.** Power of the 20-24Hz band of field potentials at 45-50 msec after light onset. **B.** Phase of the 20-24Hz band at 45-50 msec after light onset. The third column labelled K.O. shows the information found in responses to blue light in melanopsin knock out mice (396 channels, 17 animals). Since they genetically lack melanopsin they are used to confirm the amount of visual information that is lacked when melanopsin is not activated.

The main conclusion is that in different spectrotemporal components of field responses the presence of melanopsin information was confirmed and quantified for both power and phase. Information in response to blue light was significantly higher than red (Fig. 1). Melanopsin influence on the phase of field oscillations was stronger than field power. The results were confirmed in mice that genetically lacked melanopsin photoreceptors [1] (Fig. 1). This confirms that LGN receives visual content through functional pathways that are specifically sourced from melanopsin photoreceptors.
